# Retraction: *Lactobacillus gasseri* SF1183 Affects Intestinal Epithelial Cell Survival and Growth

**DOI:** 10.1371/journal.pone.0230791

**Published:** 2020-03-17

**Authors:** The PLOS ONE Editors

Following the publication of this article [1], concerns were raised regarding Figs 2, 4, and 5:

- Fig 2A, β-actin panel: the bands in lanes 3 and 4 appear similar to the bands in lanes 8 and 9 respectively.
- Figs 2B and 2D, β-actin panels: the bands in lanes 2–8 of Fig 2B are identical to the bands in lanes 1–7 of Fig 2D. The authors explained that this was the result of incorrect panel placement during the preparation of the figure and that the β-actin panel in Fig 2D is incorrect.
- Figs 2B and 2D, β-actin panels: an irregular signal was detected between the bands in lanes 5 and 6 of Fig 2B, as well as between the bands in lane 4 and 5 of Fig 2D. The authors explained that this was the result of incorrect panel placement during the preparation of the figure.
- Fig 4B, Erks ½ panel: the bands in lanes 1, 3, and 4 appear similar. The authors stand by the western blot data reported in the figure as accurate.
- Fig 5B, p21 panel: there appears to be a vertical irregularity in the background between lanes 3 and 4 suggestive of gel splicing. The authors explained that the discontinuations might be the result of imperfections in the image.

The original data for the figures of concern are no longer available. Replication data have been provided in support of the results presented in these panels; however, these data did not resolve the concerns about western blot data reporting in the original figures.

In light of the concerns affecting multiple figure panels that question the integrity of these data, the *PLOS ONE* Editors retract this article.

EC agreed with the retraction. BDL, NM, LB, VC, ER, and AP did not agree with the retraction and stand behind the published results.

## References

[1] Di Luccia B, Manzo N, Baccigalupi L, Calabrò V, Crescenzi E, Ricca E, et al (2013) *Lactobacillus gasseri* SF1183 Affects Intestinal Epithelial Cell Survival and Growth. PLoS ONE 8(7): e69102 10.1371/journal.pone.0069102 23894414 PMC3720908

